# LSD-DETR: A Lightweight Transformer-Based Detector for Sewer-Pipeline Defects in Forward-Looking Sonar Images

**DOI:** 10.3390/s26185848

**Published:** 2026-09-15

**Authors:** Yao Huang, Qingbang Han, Jinhuan Wang, Kao Ge, Nana Su, Jiayu Xia

**Affiliations:** 1 College of Information Science and Engineering, Hohai University, Nanjing 211106, China; 241313010001@hhu.edu.cn (Y.H.); 231323010012@hhu.edu.cn (J.X.); 2 College of Artificial Intelligence and Automation, Hohai University, Nanjing 211106, China; 240214010005@hhu.edu.cn (J.W.); 230222010002@hhu.edu.cn (K.G.); 3 School of Electronic and Information Engineering, Jinling Institute of Technology, Nanjing 211169, China; suyoung17@163.com

**Keywords:** underground sewer pipelines, forward-looking sonar, defect detection, lightweight detector

## Abstract

The regular inspection of underground sewer pipelines is essential for maintaining their functionality and supporting sustainable urban development. While optical sensors lack reliability, sonar imagery typically suffers from low contrast and severe noise, which is compounded by the constrained computational resources of remotely operated vehicles (ROVs). To address these limitations, this paper develops LSD-DETR, which is a lightweight Transformer-based detector for sewer pipe defects that balances accuracy and computational efficiency. First, Sonar-DualNet organizes CSP-based and HGNetV2-based blocks into a shared-stem heterogeneous backbone to reduce duplicated shallow computation while preserving complementary representations. Second, DR-Fusion performs content-adaptive modulation and aggregation of the two heterogeneous branches. In addition, the original CCFM and AIFI modules are replaced by a GLSA-enhanced BiFPN and the HiLo attention mechanism, respectively, as task-oriented adaptations for efficient cross-scale and local–global feature modeling. Experiments on the UPSD dataset show that LSD-DETR achieves an mAP50 of 89.6% and an mAP50:95 of 54.3%. Relative to RT-DETR-R18, these results represent improvements of 2.0 and 1.6 percentage points, respectively, while reducing the parameter count by 42.7% and FLOPs by 58.4%. The results demonstrate a favorable accuracy–complexity trade-off for forward-looking sonar defect detection in underground sewer pipelines.

## 1. Introduction

Underground sewer pipelines are essential components of urban drainage infrastructure and play a critical role in sewage transport, flood prevention, and environmental sanitation [1]. During long-term operation, groundwater fluctuations, soil loads, material aging, and other environmental factors can cause structural and functional deterioration [2]. Common defects include joint dislocation, leakage, sediment accumulation, root intrusion, and blockages. These defects can reduce hydraulic capacity and compromise the reliability of urban drainage systems. Periodic inspection and preventive maintenance are therefore necessary to ensure safe and effective pipeline operation [3].

Underground pipelines are enclosed, poorly illuminated, and may contain hazardous gases, making manual inspection difficult and potentially dangerous [4]. Advances in remotely operated vehicles (ROVs) and autonomous underwater vehicles (AUVs) have enabled a more automated inspection of underwater and confined structures [5,6,7]. However, in water-filled pipelines with high turbidity, optical imaging is strongly affected by light attenuation, scattering, and suspended particles. Forward-looking sonar (FLS), which uses acoustic rather than optical propagation, can operate effectively in dark and highly turbid environments. Sonar images, however, often suffer from severe speckle noise, low contrast, blurred target boundaries, and weak structural details [8]. Sonar inspection data still require substantial manual interpretation, making the process time consuming and dependent on operator experience [9]. These challenges create a clear need for accurate, automated sonar-based defect detection in underground sewer inspection.

Machine-learning and deep-learning techniques have increasingly been applied to automated pipeline condition assessment. Earlier machine-learning approaches relied on physical parameters or manually designed features [10,11], which limited their ability to capture complex defect characteristics. Deep neural networks later enabled more effective end-to-end feature learning. Two-stage detectors generally achieve high localization accuracy, whereas one-stage architectures offer greater inference efficiency. In sewer-pipeline inspection, Faster R-CNN-based frameworks have achieved promising defect-recognition performance but at relatively high computational cost [12], while YOLO-based methods allow for a faster processing of CCTV data. More recent studies have focused on improving feature extraction and multi-scale representation. For example, Guo [13] introduced an extended feature pyramid network to suppress image noise and enhance salient features. Although this approach improves detection performance, it also increases computational and storage costs. Han et al. [14] introduced the SSGD benchmark for smartphone screen-glass defects, in which targets vary substantially in size and shape. Their evaluation of CNN- and Transformer-based detectors also demonstrates the computational demands of high-resolution industrial inspection. Overall, these studies show that practical defect detection requires effective multi-scale representation while maintaining a suitable balance between detection accuracy and computational efficiency.

To address localized defects, the adaptive feature fusion U-network (AFFU) [15] selectively retains informative features. Furthermore, MSDN [16] enhances the utilization of multi-scale features, while DCCM-Net [17] facilitates the rapid and accurate recognition of road cracks. Attentional Feature Fusion (AFF) replaces fixed addition or concatenation with input-dependent attentional weighting and uses multi-scale channel attention to integrate features from different layers or branches [18]. In the context of drainage pipeline inspections, a model based on YOLOv5 [19] identifies ten common defect categories with an mAP of 83.6% at 69.9 frames per second, demonstrating substantial practical potential. However, architectures based on the YOLO framework typically generate numerous redundant bounding boxes that require non-maximum suppression (NMS). This post-processing step introduces additional computational overhead and can lead to missed detections or false positives, thereby restricting further improvements in detection speed and accuracy.

Motivated by this accuracy–efficiency requirement, Transformer-based object detectors provide an attractive direction for end-to-end inspection. Transformer-based object detectors have further advanced end-to-end detection. DETR [20] formulates object detection as a set-prediction problem and eliminates hand-crafted components such as anchors and non-maximum suppression. However, its high computational cost and slow convergence restrict real-time deployment. RT-DETR [21] improves the efficiency of the Transformer detection paradigm through an efficient hybrid encoder and optimized query-selection strategy, providing a more favorable balance between detection accuracy and inference efficiency. These characteristics make RT-DETR a suitable baseline for automated sewer inspection. However, its generic feature-extraction and aggregation architecture is not specifically designed for the weak, noisy, and scale-varying representations found in forward-looking sonar imagery.

Although progress has been made in defect recognition for underground sewer pipelines, existing methods predominantly rely on visible-light imagery under conventional conditions, leaving sonar-based detection underexplored. For forward-looking sonar sewer inspection, three coupled requirements are particularly important. First, weak local defect responses and larger structural patterns must be represented without duplicating computationally expensive high-resolution processing. Second, heterogeneous feature responses should be integrated adaptively because their relative usefulness varies across samples, channels, and spatial locations. Third, substantial defect-scale variation requires efficient cross-scale and local–global contextual modeling. Based on these considerations, we develop LSD-DETR from RT-DETR-R18. Sonar-DualNet shares shallow high-resolution processing before employing heterogeneous CSP/C2f-based and HGNetV2-based paths; DR-Fusion adaptively integrates their stage-aligned representations; GLSA-enhanced BiFPN strengthens cross-scale feature interaction; and HiLo attention provides efficient local-window and pooled-global contextual modeling. This stage-wise design targets a favorable balance between detection accuracy and computational complexity for forward-looking sonar defect detection. To distinguish the paper-specific methodological designs from components adopted from prior work, the contributions of this paper are summarized as follows:

1. We develop Sonar-DualNet, which is a shared-stem heterogeneous backbone for forward-looking sonar defect detection. Rather than introducing new convolutional operators, its paper-specific design lies in sharing the computationally expensive shallow high-resolution processing and subsequently organizing CSP/C2f-based and HGNetV2-based stages into two heterogeneous paths. This topology reduces duplicated shallow computation while retaining complementary feature transformations under a constrained computational budget.
2. We introduce DR-Fusion for the adaptive integration of stage-aligned heterogeneous backbone features. The two branch features are jointly used to generate independent spatio-channel gates and are further modulated by branch-specific, spatially shared channel-scaling vectors. Unlike normalized branch competition, the two gates are not constrained to sum to one, allowing the heterogeneous responses to be modulated independently according to the joint input representation.
3. We construct a task-oriented RT-DETR adaptation for sonar defect detection. Specifically, the original CCFM neck is replaced by a GLSA-enhanced BiFPN, and the AIFI block is replaced by the HiLo attention mechanism. Their role in LSD-DETR is to provide efficient bidirectional cross-scale interaction and complementary local–global contextual modeling within the proposed detection system.

## 2. Related Work

### Underwater Sonar Image Object Detection

Underwater sonar object detection has gradually shifted from hand-crafted feature extraction to deep-learning-based end-to-end frameworks. Traditional methods typically rely on threshold segmentation, shadow analysis, texture descriptors, or region proposals, but their performance is sensitive to speckle noise, background clutter, imaging-angle variation, and target-scale changes [22,23]. CNN-based detectors such as Faster R-CNN, SSD, and YOLO have subsequently been introduced into side-scan sonar (SSS) and forward-looking sonar (FLS) applications, substantially improving feature representation and detection robustness. Recent reviews indicate that YOLO-based architectures are particularly common because of their favorable balance between detection accuracy and inference efficiency [24,25].

Recent studies’ SSS detection have mainly focused on feature enhancement, lightweight network design, and weak-target representation. Wen et al. [26] incorporated the Swin Transformer, CBAM attention, and feature scaling into YOLOv7 to improve detection under blurred details and complex backgrounds. Gao et al. [27] proposed GCT-YOLOv5, a lightweight detector designed for real-time SSS processing, demonstrating the practical benefits of efficient network design. Zhang et al. [28] further developed ESL-YOLO by introducing edge-aware modeling and adaptive image-quality assessment to better preserve weak boundaries and low-contrast target information in sonar images.

Forward-looking sonar (FLS) detection places greater emphasis on noise robustness, spatial semantic representation, and multi-scale feature extraction. Xu et al. [29] proposed AquaPile-YOLO for underwater pile-foundation inspection using FLS imagery. Ge et al. [30] developed a multibeam FLS detection framework that uses multi-scale feature extraction and feature re-weighting to improve weak-target recognition. More recently, Wang et al. [31] investigated noise-robust object detection in FLS imagery, showing that effective detection requires suppressing background interference without weakening target responses. These studies suggest that efficient feature extraction, weak-target representation, multi-scale modeling, and noise robustness remain key challenges in sonar object detection. In sewer-pipeline FLS imagery, these challenges are further complicated by limited computational resources and complex structural backgrounds.

Data scarcity and domain variation across sonar platforms have also motivated research on transfer learning, data augmentation, and robust representation learning [32]. Although these approaches can improve model adaptability, the reliable detection of weak targets under strong noise and limited computational resources remains an open problem.

Overall, existing sonar detectors have achieved substantial progress through attention mechanisms, lightweight backbones, and multi-scale feature enhancement. Nevertheless, jointly maintaining representation diversity, adaptive feature integration, and computational efficiency remains challenging for forward-looking sonar inspection in confined sewer environments. These observations motivate the heterogeneous and efficiency-oriented architecture introduced in the following section.

## 3. Method

### 3.1. Overall Structure of the Model

**Figure 1:**
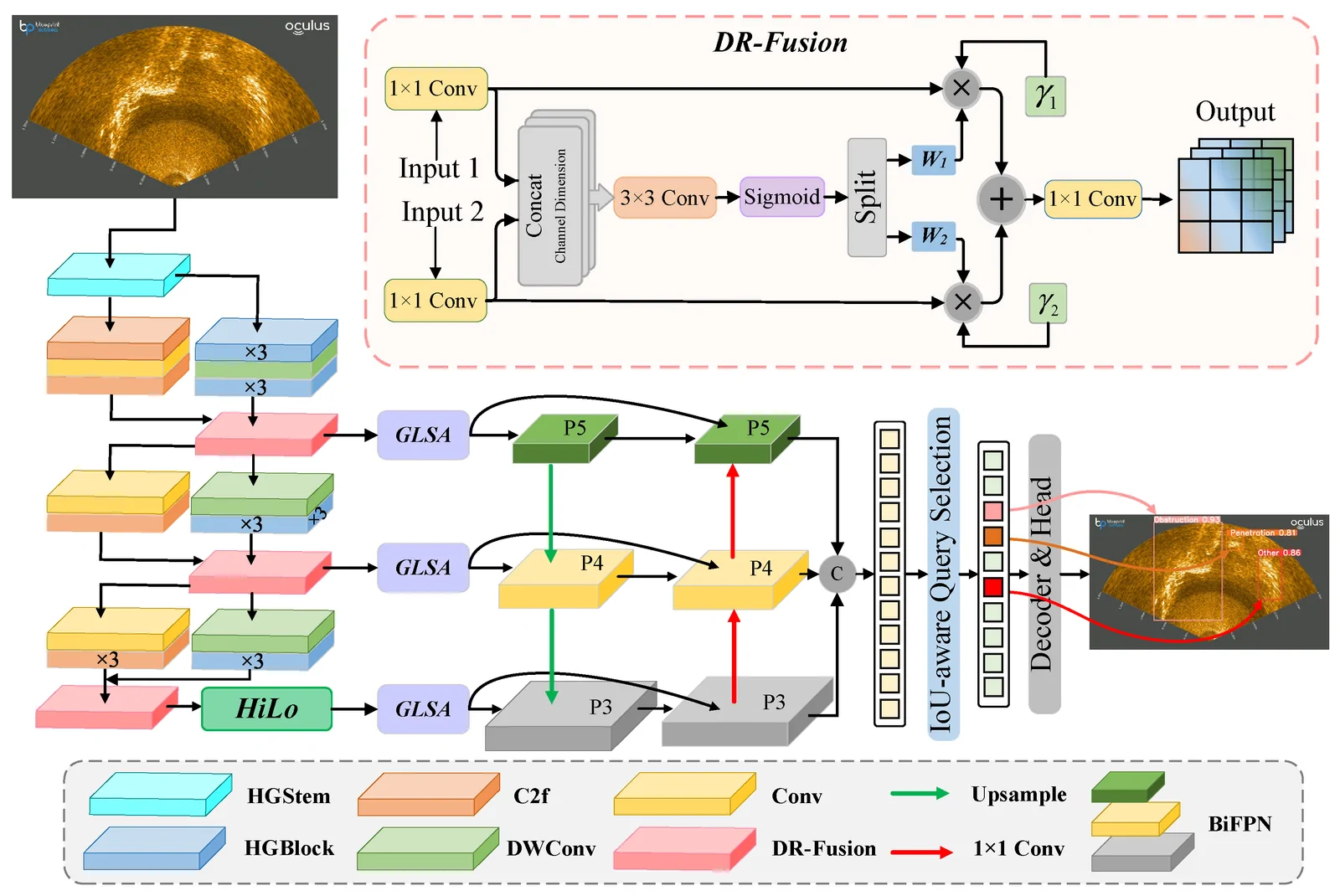
Overall architecture of LSD-DETR. The proposed architecture employs Sonar-DualNet, integrated with the DR-Fusion module, as the backbone network. The extracted features are subsequently processed by the GLSA-BiFPN neck structure and a decoder equipped with an IoU-aware query selection mechanism to generate the final detection results.

The proposed architecture, as illustrated in Figure 1, integrates several specialized modules to address the challenges specific to the sonar-based detection of pipeline defects. RT-DETR-R18 is adopted as the baseline detector. The IoU-aware query selection strategy, Transformer decoder, matching procedure, and detection head are retained from the original RT-DETR framework without modification.

The proposed changes are restricted to three components: (1) replacing the ResNet-18 backbone with Sonar-DualNet, (2) replacing the original CCFM neck with a GLSA-enhanced BiFPN, and (3) replacing AIFI with HiLo attention. Within the backbone, Sonar-DualNet employs a shared HGStem to reduce computational overhead during the shallow, high-resolution processing stages before branching into two heterogeneous paths. To integrate the complementary representations, DR-Fusion first projects the two branch features to a common channel dimension. It then generates two independent input-dependent spatio-channel gates from their concatenated representation and combines them with spatially shared channel-scaling coefficients before aggregation.

In the neck module, GLSA-BiFPN extends the bidirectional cross-scale transmission of BiFPN by embedding the global–local dual-stream attention mechanism of GLSA at each scale. This integration enables the simultaneous perception of the macroscopic structures of the pipeline and the local textures of defects, thereby improving the recognition of ill-defined boundaries and densely distributed targets. Finally, the encoder employs HiLo attention, which divides the attention heads between a local-window Hi-Fi branch and a pooled-global Lo-Fi branch. These two paths model fine-grained local details and large-scale contextual relationships, respectively, while reducing the computational cost of global self-attention. Collectively, these components form a compact detection architecture that balances detection accuracy with parameter and FLOP efficiency.

### 3.2. Sonar-DualNet

**Figure 2:**
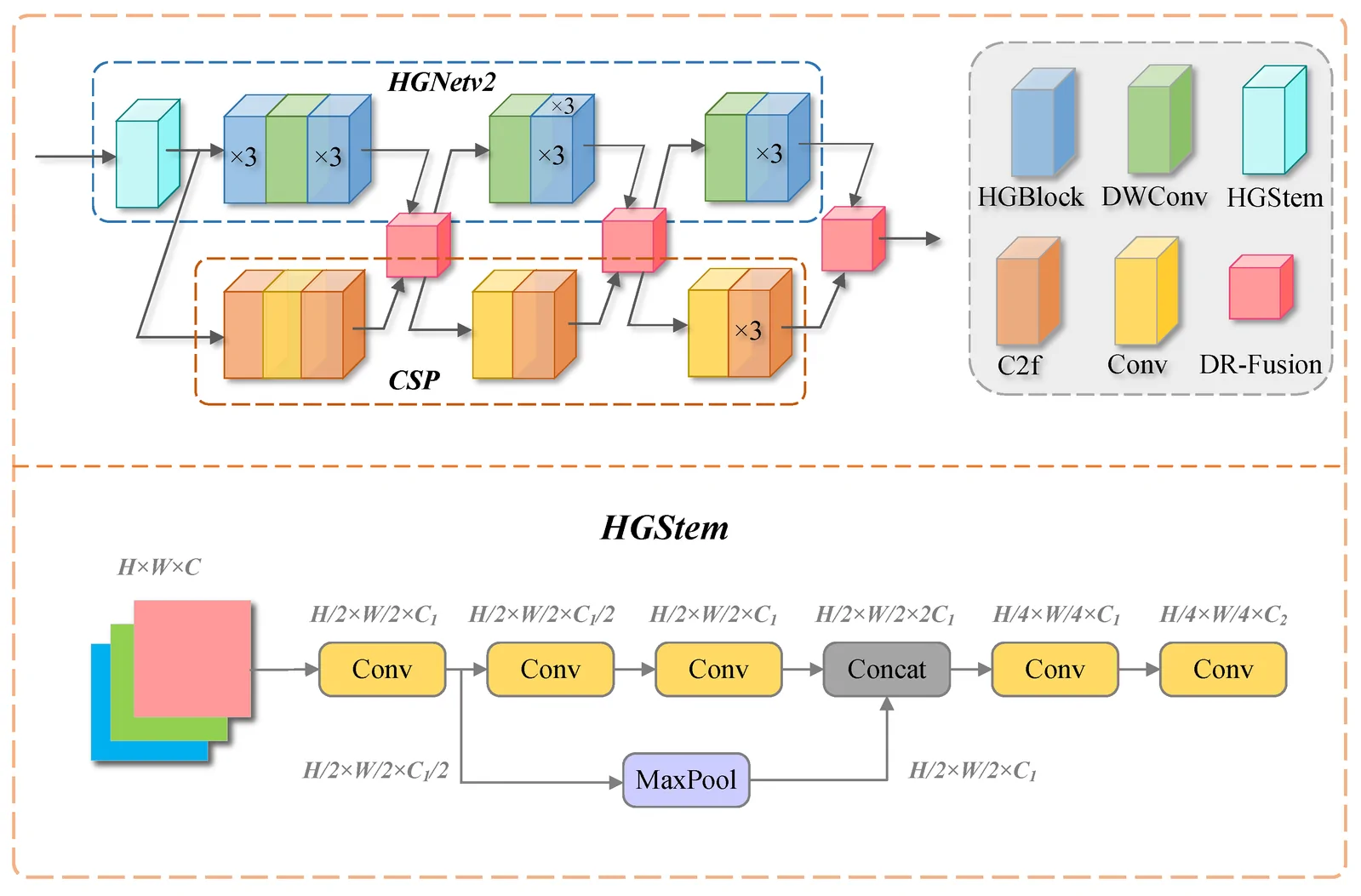
Detailed structure of the Sonar-DualNet backbone.

Current backbone networks for object detection are typically optimized for natural optical images, inherently incurring substantial parameter counts and computational redundancy. Directly applying these architectures to the sonar-based inspection of pipelines yields suboptimal results, as they fail to capture the specific characteristics of sonar imagery and incur substantial parameter and computational costs. To address these limitations, we propose Sonar-DualNet, a dual-path backbone architecture, as illustrated in Figure 2.

By employing two lightweight, complementary parallel branches, the proposed approach efficiently extracts multi-level features from sonar images while strictly constraining the overall parameter count.

In deep convolutional neural networks, shallow feature extraction typically accounts for a substantial proportion of FLOPs and inference time due to high input resolutions. To reduce this computational burden without sacrificing feature diversity, we design a shared HGStem module as the initial stage of the backbone network. As illustrated in Figure 2, the HGStem module consists of five convolutional layers and one max pooling layer.

The input sonar image, denoted by X, initially passes through a 3×3 convolutional layer with a stride of two for rapid downsampling, producing the initial feature map $F_{0}$, which is formulated as

(1) $$F_{0}=\mathrm{ReLU}\left(\mathrm{BN}\left(\mathrm{Conv}\left(X\right)\right)\right).$$

Subsequent lightweight convolutions and multi-path aggregation capture low-level spatial information with limited computational overhead. Because the stem features are shared, the following CSP [33] and HGNetV2 [21] branches avoid redundant processing at high resolutions. This design reduces repeated computation on shallow high-resolution feature maps and lowers the overall parameter count and FLOPs.

After the shared HGStem, the network splits into two heterogeneous branches that capture different characteristics of sonar defects. Built on the C2f module, the CSP branch divides the feature map along the channel dimension and progressively extracts features through lightweight bottlenecks. The resulting features are concatenated to retain gradient information while limiting parameter growth and feature redundancy. This structure is well suited to continuous geometric defects, such as elongated cracks. In contrast, the HGNetV2 branch uses HGBlocks for hierarchical feature interaction and deep feature fusion. It captures higher-level semantic information and provides stronger discrimination for the diffuse defects caused by acoustic scattering, such as corrosion and deposition. Together, the two branches retain shallow spatial details and deep semantic information without substantially increasing model complexity.

Sonar-DualNet does not introduce new C2f, HGBlock, or HGStem operators. Its paper-specific design lies in organizing these existing operators into a shared-stem heterogeneous topology. The computationally expensive shallow high-resolution processing is shared by the two subsequent paths, after which CSP/C2f-based and HGNetV2-based stages provide different feature-transformation pathways. Their stage-aligned representations are subsequently integrated through DR-Fusion. This organization is intended to balance computational reuse and representation diversity rather than simply increasing the number of parallel branches.

### 3.3. DR-Fusion

The CSP-based and HGNetV2-based paths employ different feature-transformation operators and consequently produce heterogeneous responses. Direct element-wise addition assigns the same aggregation rule to both paths, whereas simple concatenation relies entirely on the subsequent convolution to select useful information. Neither strategy explicitly performs input-conditioned modulation before aggregation. This issue is relevant to sonar imagery because the relative usefulness of local texture responses and larger structural patterns may vary across samples, channels, and spatial locations. We therefore introduce DR-Fusion to recalibrate the two heterogeneous representations before fusion.

**Figure 3:**
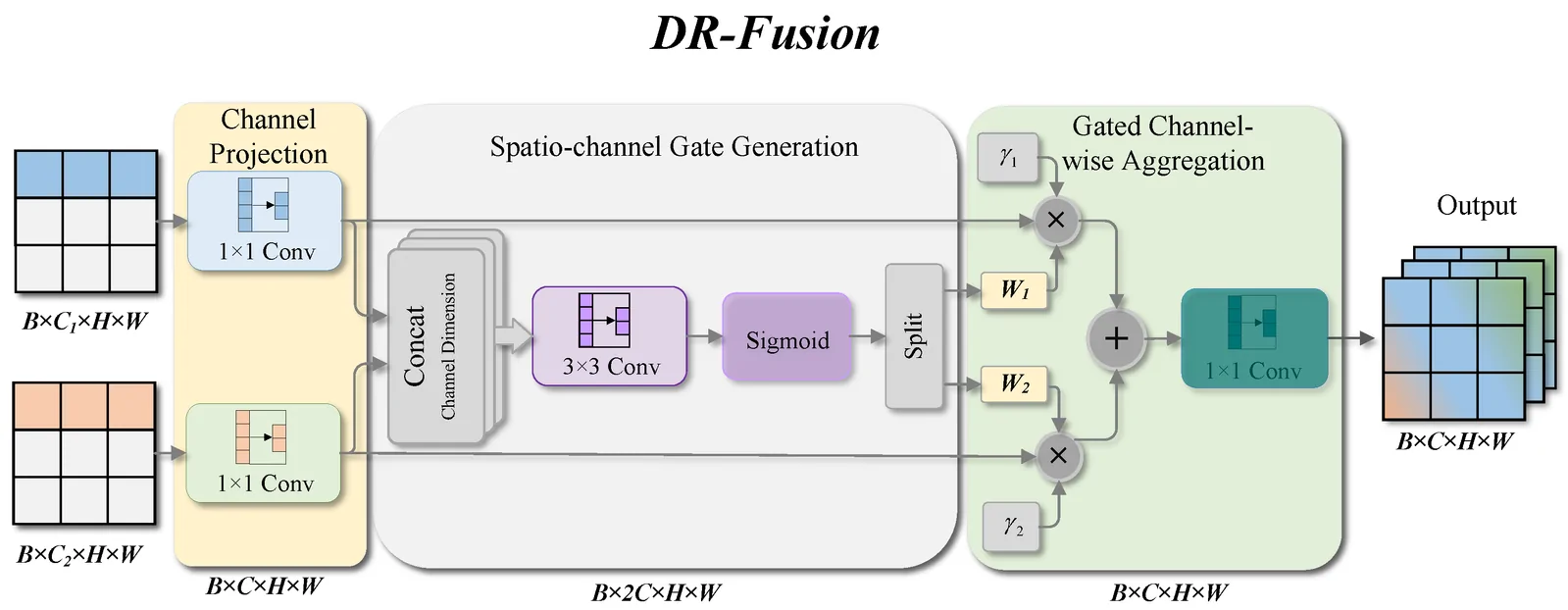
Architecture of DR-Fusion.

To address this limitation, we propose a dynamic recalibration fusion module, which is denoted as DR-Fusion. As illustrated in the lower section of Figure 3, this process involves channel projection, spatio-channel gate generation, and gated channel-wise aggregation.

Let the two input feature tensors be $X_{1}\in R^{B\times C_{1}\times H\times W}$, $X_{2}\in R^{B\times C_{2}\times H\times W}$. DR-Fusion is applied to feature maps from corresponding backbone stages, and the two inputs therefore have the same spatial resolution. Two independent 1×1 convolution blocks project them to a common channel dimension *C*:

(2) $${\bar{X}}_{1}=\phi_{1}\left(X_{1}\right),\;{\bar{X}}_{2}=\phi_{2}\left(X_{2}\right),$$

where ${\bar{X}}_{1},{\bar{X}}_{2}\in R^{B\times C\times H\times W}$. This operation aligns only the channel dimensions; DR-Fusion does not perform geometric registration or spatial-resolution transformation.

The aligned features are concatenated and processed by a 3×3 convolution block with 2C output channels. An element-wise sigmoid function is then applied:

(3) $$Z=\sigma \left(\psi_{3\times 3}\left([{\bar{X}}_{1};{\bar{X}}_{2}]\right)\right),Z\in {\left(0,1\right)}^{B\times 2C\times H\times W},$$

the output is divided equally along the channel dimension:

(4) $$\left[W_{1},W_{2}\right]=\mathrm{Split}\left(Z\right),W_{1},W_{2}\in {\left(0,1\right)}^{B\times C\times H\times W},$$

here, [·;·] denotes channel-wise concatenation. Each gate is conditioned jointly on the two branch features and modulates one branch at every channel and spatial position. Because the two gates are produced by independent sigmoid outputs rather than branch-wise softmax normalization, $W_{1}+W_{2}=1$ is not enforced. They are therefore interpreted as independent spatio-channel gates rather than normalized competitive weights. DR-Fusion further introduces two branch-specific channel-scaling tensors: $\gamma_{1},\gamma_{2}\in R^{1\times C\times 1\times 1}$. They are initialized to 0.5 and clipped element-wise to [−1,1] before each forward pass:

(5) $$\gamma_{i}\leftarrow \mathrm{clip}\left(\gamma_{i},-1,1\right),\;i\in \left\{1,2\right\}.$$

The tensors are shared across samples and spatial positions but are learned independently for each channel. The final fused representation is computed as

(6) $$Y=\phi_{o}\left(W_{1}⊙{\bar{X}}_{1}⊙\gamma_{1}+W_{2}⊙{\bar{X}}_{2}⊙\gamma_{2}\right),$$

where $\phi_{o}$ denotes the final 1×1 convolution block and ⊙ denotes element-wise multiplication with broadcasting.

DR-Fusion is specifically designed for the stage-aligned heterogeneous representations produced by Sonar-DualNet. Its key design is a dual-level recalibration mechanism that combines jointly conditioned, independent spatio-channel gating with branch-specific, spatially shared channel scaling. Because the two gates are generated independently rather than through branch-wise Softmax normalization, DR-Fusion does not impose a unit-sum competition between the heterogeneous branches, enabling both representations to be enhanced or suppressed according to their input-dependent relevance.

### 3.4. GLSA-BiFPN

**Figure 4:**
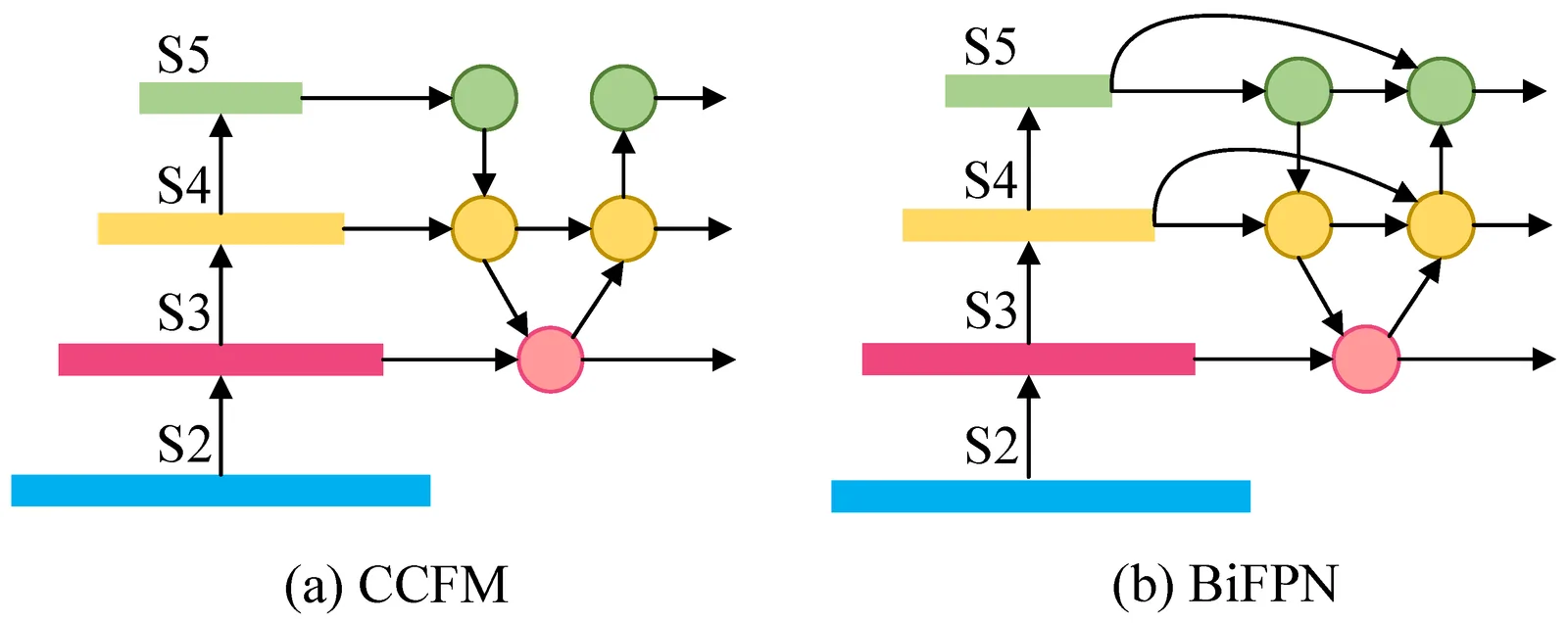
Overview of pyramid feature fusion networks. (**a**) CCFM adopts an inverted-pyramid strategy; (**b**) BiFPN performs bidirectional fusion with learnable weights.

**Figure 5:**
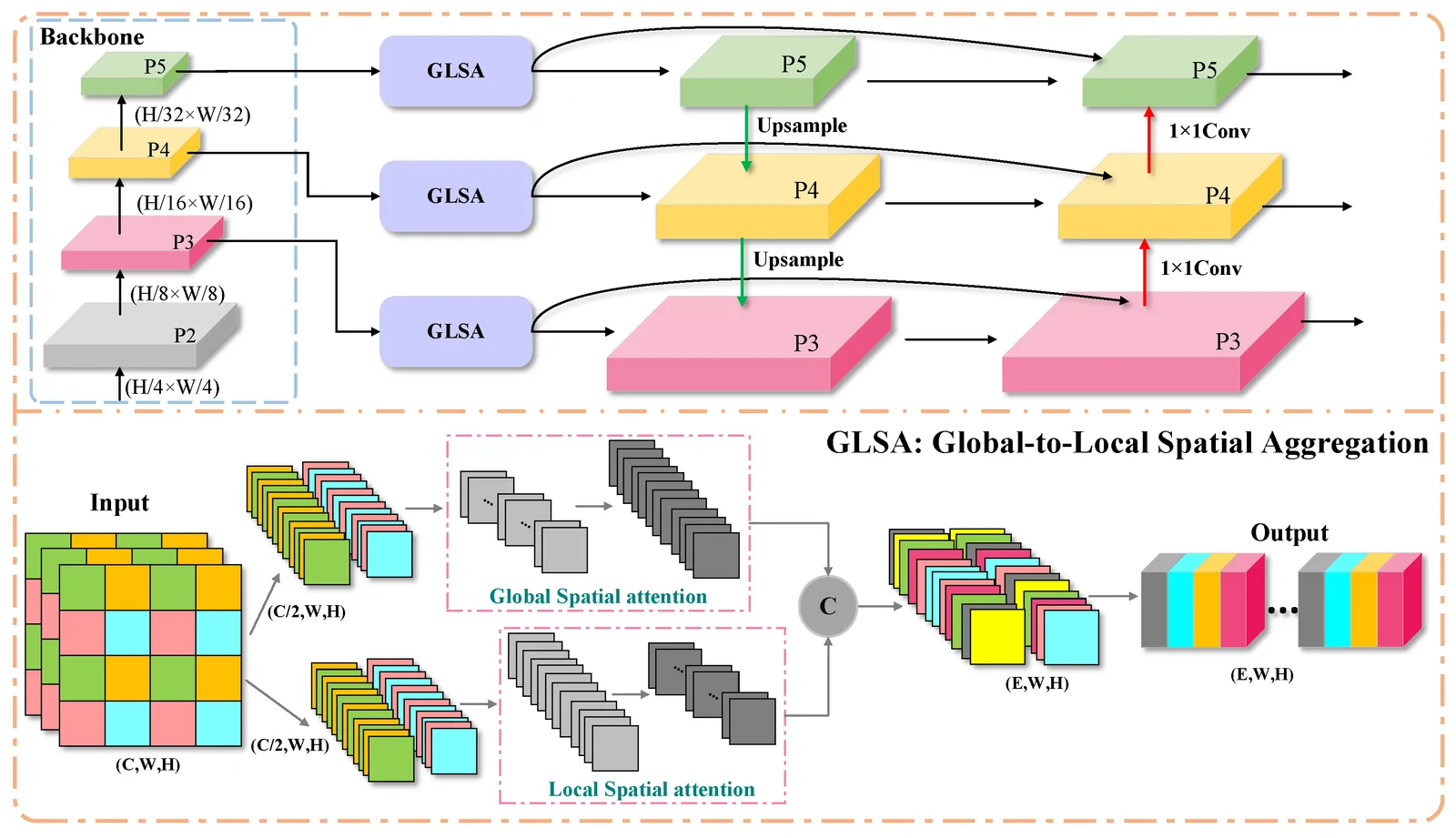
Structure of GLSA-BiFPN.

The original neck architecture of RT-DETR, as illustrated in Figure 4a, employs the CCFM module to fuse multi-scale features. As this convolutional stacking mechanism lacks any explicit modeling of cross-scale contributions, it demonstrates difficulty in balancing the fine-grained localization of small defects and the global perception of large structures within pipelines. To address this limitation, we replace the CCFM module with the GLSA-BiFPN architecture, as depicted in Figure 5. This design preserves the bidirectional flow of multi-scale features while facilitating the targeted enhancement of spatial details at each representation level. We retain the normalized weighted fusion rule of BiFPN [34] and the original GSA/LSA dual-branch formulation of GLSA [35].

Traditional FPN performs multi-scale feature aggregation through a top–down pathway, where high-level semantic features are progressively propagated to lower layers by upsampling. Although this strategy enhances semantic representation, its one-way information flow can weaken the preservation of spatial details in low-level features, which is particularly unfavorable for sonar target detection that relies heavily on subtle texture cues. To improve feature representation across different scales, an enhanced Bidirectional Feature Pyramid Network (BiFPN) is utilized, enabling bidirectional information exchange and more effective interaction among multi-level features.

This design maintains detailed spatial information from shallow features while strengthening the semantic guidance provided by deeper layers for small-target representation. As shown in Figure 5, the modified GLSA-BiFPN improves the model’s ability to capture subtle defect textures and enhances detection accuracy. The proposed structure mainly comprises feature selection and multi-scale fusion stages. Since high-level feature channels contribute relatively limited information to fine defect localization, greater emphasis is assigned to the middle- and low-level representations. In particular, feature maps generated at different backbone stages are first subjected to channel selection at the lower levels, and the retained features are subsequently delivered to a multi-level enhancement module for joint fusion. Through this process, contextual cues from different scales are aggregated more effectively, while high-level semantics are incorporated into detail-rich low-level features, thereby improving the detection of densely distributed small defects.

#### GLSA

**Figure 6:**
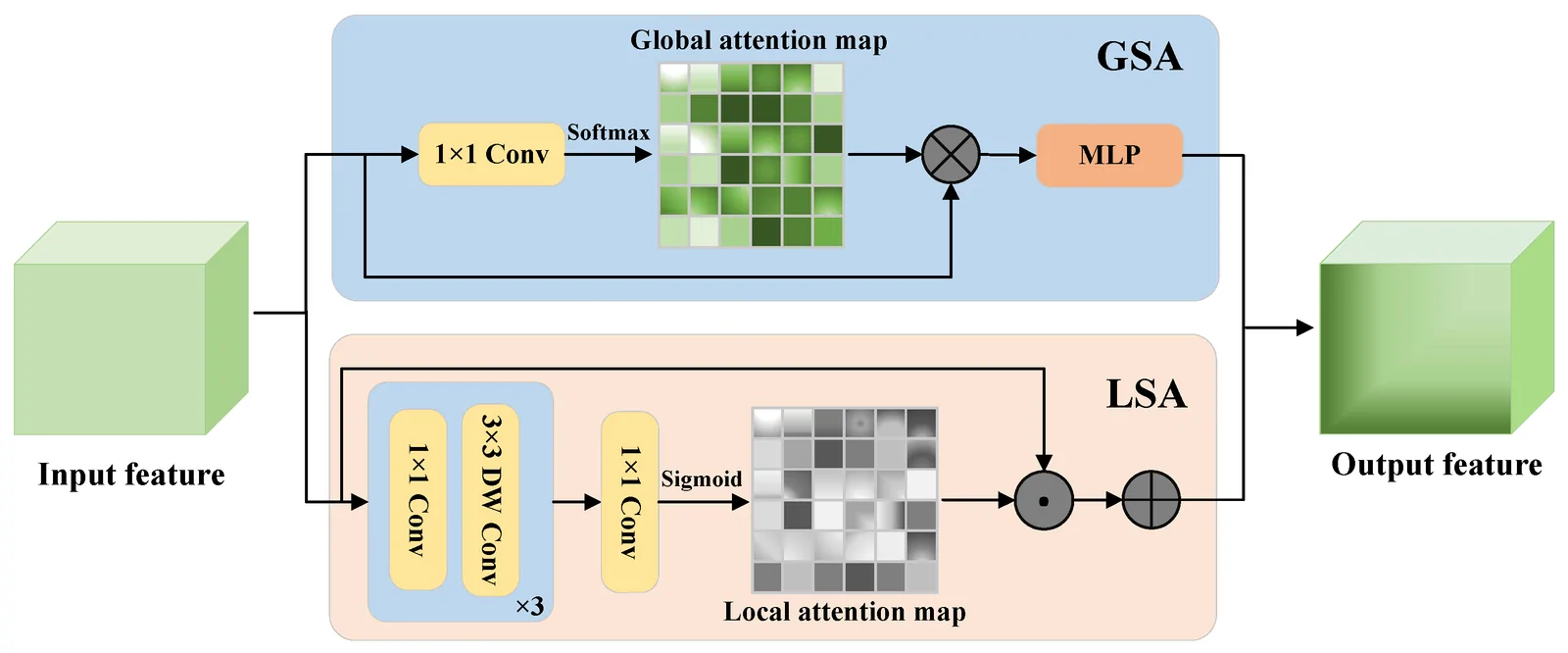
Overview of the global-to-local spatial aggregation (GLSA) module, which consists of global spatial attention (GSA) and local spatial attention (LSA).

As illustrated in Figure 6, the global–local spatial awareness (GLSA) module partitions the input feature map into two equal channel groups, which are processed in parallel by the GSA and LSA branches. The parallel outputs are subsequently concatenated and fused through a 1×1 convolution:

(7) $$F_{i}^{\prime }={\mathrm{Conv}}_{1\times 1}\left[\;\mathrm{GSA}\;\left(F_{i}^{1}\right);\;\mathrm{LSA}\;\left(F_{i}^{2}\right)\;\right],$$

where $F_{i}^{1}$ and $F_{i}^{2}$ denote the channel-split sub-features at the *i*-th scale, and [·;·] denotes channel-wise concatenation.

As depicted in Figure 6, in contrast to local convolutions, the GSA branch facilitates direct interactions among all spatial locations via matrix multiplication. This mechanism captures cross-regional structural correlations, including pipeline orientations and bending deformations, without introducing positional bias, thereby providing a global semantic context for the subsequent localization of defects.

In contrast, the LSA branch stacks three basic units comprising 1×1 and 3×3 depthwise separable convolutions. This branch generates a spatial attention mask via a sigmoid activation function, which is multiplied element-wise with the input to capture high-frequency local features, such as the textures of cracks and the misalignments of joints. The attention mechanism within the LSA branch is formulated as

(8) $$\mathrm{LSA}\left(F_{i}^{2}\right)=\sigma \;\left({\mathrm{Conv}}_{1\times 1}\left(F_{c}\left(F_{i}^{2}\right)\right)\right)⊙F_{i}^{2}+F_{i}^{2},$$

where $F_{c}\left(\cdot \right)$ represents the cascaded convolutions, σ(·) denotes the sigmoid activation function, and ⊙ indicates element-wise multiplication.

Ultimately, the BiFPN architecture performs adaptive weighted fusion across multi-scale feature layers, whereas the GLSA module complementarily enhances global semantics and local details within each scale. Collectively, these components constitute an efficient feature neck optimized for the sonar-based detection of pipeline defects.

### 3.5. HiLo Module

Within Vision Transformers, standard multi-head self-attention (MSA) computes global attention across all image patches, resulting in a computational complexity of $O(N^{2}C)$, where *N* represents the sequence length and *C* denotes the feature dimension. For high-resolution feature maps, this quadratic overhead severely constrains detection speed. This bottleneck is particularly relevant to sonar-based pipeline inspection, where useful defect representations involve both fine-grained local patterns, such as crack textures and joint discontinuities, and large-scale structural context, such as the overall pipeline contour.

Standard global MSA processes all tokens uniformly and incurs quadratic computational cost without explicitly separating efficient local interaction from compressed global-context modeling.

**Figure 7:**
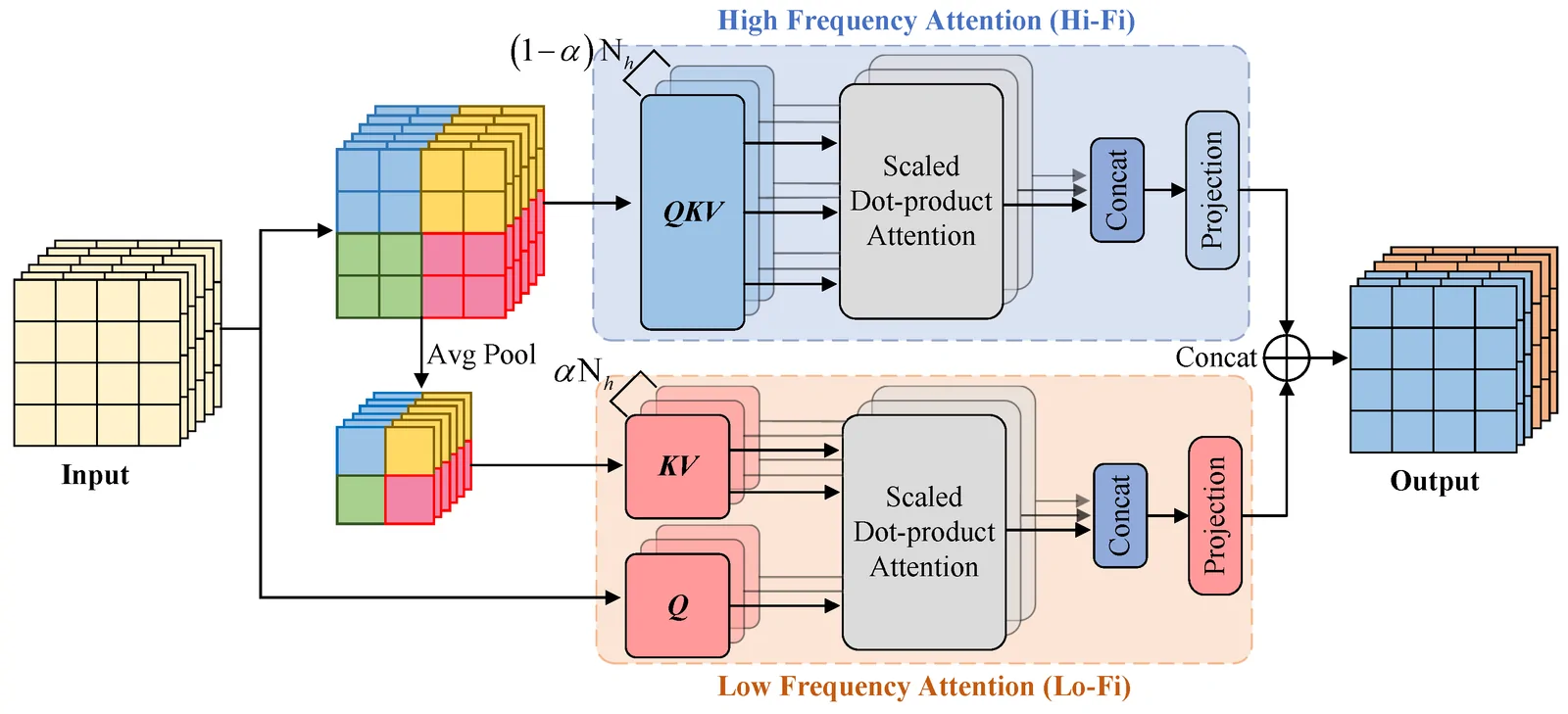
Dual-path structure of HiLo attention. The Hi-Fi branch performs local-window attention, whereas the Lo-Fi branch uses full-resolution queries and spatially pooled keys and values for global interaction.

This paper integrates the HiLo attention mechanism [36], which partitions the attention heads into two groups according to their spatial interaction ranges and computational pathways. The Hi-Fi group performs attention within non-overlapping local windows, whereas the Lo-Fi group models global interactions using full-resolution queries and spatially pooled keys and values. The corresponding schematic is illustrated in Figure 7.

Let $N_{h}$ denote the total number of attention heads, and let α∈(0,1) represent a split ratio. Based on this ratio, $\left(1-\alpha \right)N_{h}$ heads are allocated to the high-frequency attention (Hi-Fi) branch, whereas the remaining $\alpha N_{h}$ heads are assigned to the low-frequency attention (Lo-Fi) branch. The Hi-Fi branch performs standard self-attention within non-overlapping local windows of size s×s, strictly confining the computation to the window boundaries. This mechanism reduces the computational complexity of standard multi-head self-attention from $O(N^{2})$ to $O(N\cdot s^{2})$. The proposed approach therefore achieves substantial acceleration for small window sizes, such as s=2, while preserving the capacity to capture local details.

The Lo-Fi branch facilitates the efficient modeling of global structures. Applying average pooling to each local window reduces the sequence length of the keys and values from *N* to $N/s^{2}$. Concurrently, deriving queries directly from the original feature map preserves the full spatial resolution. The attention mechanism within this branch is formulated as

(9) $$\mathrm{LoFi}\left(X\right)=\mathrm{Softmax}\left(\frac{QK^{⊤}}{\sqrt{D_{h}}}\right)V,$$

where $K,V\in R^{\left(N/s^{2}\right)\times D_{h}}$ denote the downsampled key and value matrices obtained via average pooling. Spatial average pooling reduces the number of key and value tokens while preserving an aggregated representation of each local region. Consequently, the Lo-Fi branch maintains a global receptive field with substantially lower attention cost. Moreover, as the reduced sequence length $N/s^{2}$ is substantially smaller than the original length, this branch effectively decreases computational overhead while maintaining a global receptive field.

The projected outputs of the two branches are subsequently concatenated along the channel dimension to combine local-window representations with pooled-global contextual representations:

(10) $$\mathrm{HiLo}\left(X\right)=\mathrm{Concat}\left(\mathrm{HiFi}\left(X\right)W_{o}^{h},\mathrm{LoFi}\left(X\right)W_{o}^{l}\right),$$

where $W_{o}^{h}$ and $W_{o}^{l}$ denote the branch-specific output projection matrices of the Hi-Fi and Lo-Fi paths, respectively, and Concat(·) denotes concatenation along the channel dimension. Within the RT-DETR encoder, AIFI applies global self-attention to all spatial tokens. Replacing AIFI with HiLo separates the computation into local-window attention and pooled-global attention. The local branch preserves detailed interactions within small spatial regions, whereas the pooled-global branch models long-range contextual relationships using fewer key and value tokens. This design provides complementary local and global representations with reduced attention complexity.

## 4. Experimental Settings

### 4.1. Dataset Collection and Creation

This paper uses the Underground Pipe Sonar Defect (UPSD) dataset, which was collected using high-frequency forward-looking sonar in water-filled, highly turbid, and confined pipeline environments. The dataset was collected by AutoSubsea Vehicles Inc. (Shanghai, China). during real-world engineering inspections and therefore reflects practical defect detection conditions. In addition, the publicly available SCTD [37] (Sonar Common Target Detection) dataset is used as an external benchmark to evaluate the proposed model under different sonar imaging conditions and provide a broader assessment of its performance.

The UPSD dataset was acquired using an Oculus MD750D multibeam forward-looking sonar (Blueprint Subsea, Ulverston, Cumbria, UK). in water-filled and turbid sewer pipes. Detailed specifications of the sonar are provided in Table 1. During data collection, the Oculus MD750D was mounted on an ROV-based pipe-inspection platform operating inside underground sewer pipes. To reflect practical inspection conditions, in situ surveys were conducted across urban trunk drainage networks in Quanzhou, Fujian Province, China. Data collection focused on DN1200 reinforced concrete pipes, which are widely used in urban trunk drainage systems and provide a representative yet challenging setting for autonomous inspection.

**Figure 8:**
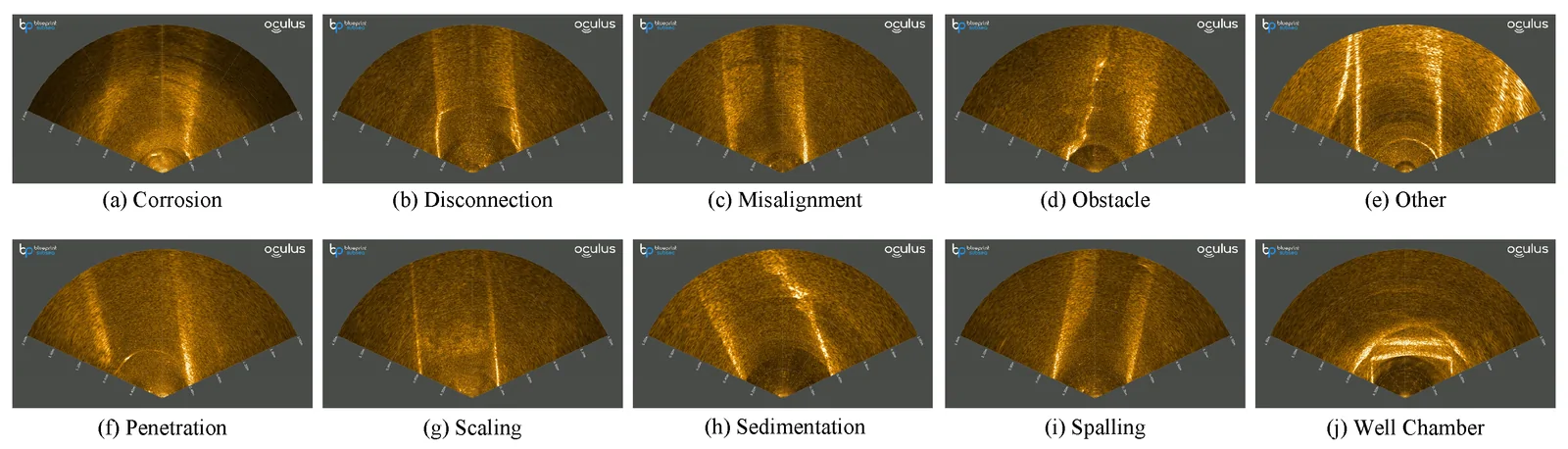
Representative examples of the ten defect categories in the UPSD dataset.

The dataset developed in this paper contains ten categories of structural and functional defects, as shown in Figure 8. These categories include Corrosion, Disconnection, Misalignment, Obstacle, Other, Penetration, Scaling, Sedimentation, Spalling, and Well Chamber. To ensure the consistent and reproducible annotation of sewer defects in sonar images, the labeling protocol was developed with reference to real engineering inspection scenarios and combines experience from optical image interpretation with the specific echo characteristics of sonar data. These ten semantic categories are used for model training and automated defect detection.

These definitions provide consistent criteria for sonar defect annotation. The raw UPSD data were collected as continuous forward-looking sonar video streams during sewer-pipeline inspections conducted using an underwater remotely operated vehicle. Individual frames were extracted from the videos, and duplicate or highly similar frames were removed. The remaining samples were manually screened and annotated using Labelme (wkentaro/labelme, GitHub) with a rectangular bounding box assigned to each defect instance. The annotations were first stored in JSON format and then converted to YOLO format for model training.

**Figure 9:**
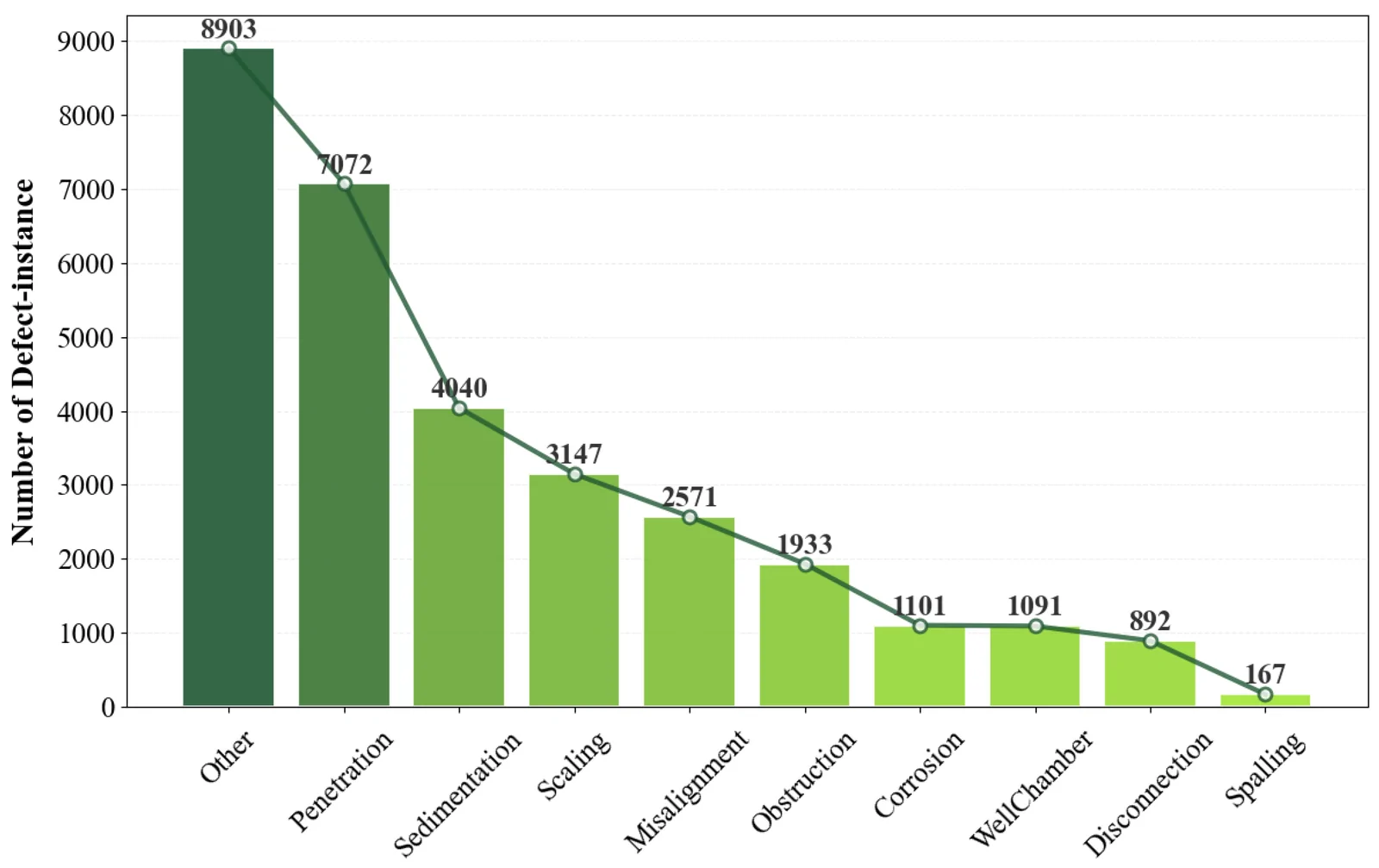
Defect-instance distribution of the UPSD dataset.

To ensure annotation quality, two domain experts independently annotated the samples and reviewed the category labels and bounding-box locations. Samples with ambiguous or inconsistent annotations were excluded, resulting in 23,819 valid sonar frames. The sample distribution is shown in Figure 9.

To avoid temporal leakage, adjacent frames containing the same defect were grouped based on their source video and temporal continuity. The dataset was then divided at the group level into training, validation, and test sets at an approximate ratio of 6:2:2, containing 14,291, 4764, and 4764 images, respectively. This grouping ensured that frames from the same defect sequence were assigned to only one subset. For computational efficiency, all images were cropped to 640×640 pixels before model training and evaluation.

The SCTD dataset is a publicly available benchmark for underwater sonar object detection. It contains several common underwater target categories, including drowning victims, airplanes, and shipwrecks, with a total of 596 annotated targets in Pascal VOC format. The dataset is divided into training, validation, and test sets containing 298, 99, and 100 images, respectively.

### 4.2. Training Environment and Detailed Settings

For a fair and reproducible comparison, all experiments were conducted using the same hardware and software environment. The experimental platform was equipped with an Intel Core i9-14900KF CPU (Intel Corporation, Santa Clara, CA, USA) running at 3.20 GHz, 24 GB of system memory, and an NVIDIA GeForce RTX 4090 GPU (NVIDIA Corporation, Santa Clara, CA, USA). The workstation ran Windows 10, version 22H2 (Microsoft Corporation, Redmond, WA, USA), CUDA 12.6 (NVIDIA Corporation, Santa Clara, CA, USA), Python 3.9 (Python Software Foundation, Wilmington, DE, USA), and PyTorch 2.1.0 (Meta Platforms, Inc., Menlo Park, CA, USA) for model implementation and training. All models are trained and evaluated using the same dataset partitions, 640×640 input resolution, hardware environment, evaluation protocol, 150 training epochs, batch size of 4, and 4 dataloader workers without using pre-trained weights. Model-specific optimization settings, including the optimizer and learning-rate schedule, follow the respective official recommendations. For LSD-DETR ablation experiments, identical training settings are used across all variants. For LSD-DETR, AdamW is employed with an initial learning rate of 0.0001, a momentum of 0.9, and a weight decay of 0.0001. The learning-rate warmup is applied during the first 2000 training iterations.

### 4.3. Evaluation Metrics

To assess the detection capability and computational efficiency of the proposed LSD-DETR, this paper adopts a set of commonly used evaluation indicators, including Mean Average Precision (mAP), Precision, Recall, Frames Per Second (FPS), parameter count (Params), and Giga Floating-point Operations (GFLOPs).

Precision measures the proportion of correct positive predictions among all identified objects. The calculation is formulated as follows:

(11) $$\mathrm{Precision}=\frac{TP}{TP+FP}.$$

Recall represents the ratio of correctly identified objects to the total number of actual objects, which is calculated as

(12) $$\mathrm{Recall}=\frac{TP}{TP+FN},$$

where TP, FP, and FN denote the number of true positives, false positives, and false negatives, respectively. Average Precision (AP) signifies the area under the Precision–Recall curve, reflecting the average maximum precision for different categories at various recall levels. It is defined as

(13) $$\mathrm{AP}=\int_{0}^{1}\mathrm{Precision}(\mathrm{Recall})d(\mathrm{Recall}).$$

Mean Average Precision computes the mean of the AP values across all categories, indicating the overall detection performance of the model on the entire dataset. This metric is computed as

(14) $$\mathrm{mAP}=\frac{1}{N}\sum_{i=1}^{N}AP_{i},$$

the metric mAP50 refers to the mAP evaluated at an Intersection over Union (IoU) threshold of 0.5. Similarly, mAP50:95 denotes the mAP evaluated over a range of IoU thresholds from 0.5 to 0.95.

FPS represents the inference throughput measured under the specified hardware, batch-size, precision, and timing protocol:

(15) $$\mathrm{FPS}=\frac{1000}{T_{\mathrm{preprocess}}+T_{\mathrm{inference}}+T_{\mathrm{postprocess}}},$$

where $T_{\mathrm{preprocess}}$, $T_{\mathrm{inference}}$, and $T_{\mathrm{postprocess}}$ denote the average per-image time required for data preprocessing, model forward inference, and prediction post-processing, respectively. All our speed measurements are conducted with a batch size of 1 to reflect the single-image processing capability required in real-time applications.

Params refers to the total number of trainable weights in the model, typically measured in millions, reflecting the structural complexity and memory consumption of the network. Finally, GFLOPs represent the number of floating-point operations required for a single forward pass, which is measured in billions. This final metric assesses the computational cost and theoretical inference efficiency of the algorithm.

## 5. Results and Discussion

### 5.1. Ablation Experiment

To validate the contributions of the proposed core modules to the overall detection framework, systematic ablation studies are conducted on the UPSD dataset, which is a custom collection of sewer sonar imagery. The quantitative results presented in Table 2 illustrate the trade-offs between computational complexity and detection accuracy for the individual modules and their combinations.

(1) Performance and Efficiency Analysis of Individual Modules

Relative to the baseline architecture, each independent modification enhances detection performance to varying degrees. Incorporating the Sonar-DualNet module alone reduces the parameter count to 13.99 M and computational overhead to 34.0 G while elevating the mAP50 to 0.886. This result indicates that sharing the HGStem to compress shallow computational operations, coupled with the heterogeneous parallel pathways of CSP and HGNetV2, effectively extracts complementary local and global features from sonar imagery while compressing the overall model.

Applying the GLSA-BiFPN module in isolation also improves the mAP50 and reduces computational complexity. This improvement demonstrates that embedding a global–local dual-stream attention mechanism within the feature pyramid increases the capacity of the model to recognize targets exhibiting ill-defined boundaries and densely distributed defects. Finally, integrating the HiLo module independently maintains a parameter count and computational overhead comparable to the baseline, yet it increases the mAP50 to 0.880. Decoupling the attention heads into high-frequency local and low-frequency global branches enhances representation specificity and inference efficiency without increasing the original computational load.

(2) Module Synergy and Overall Architectural Advantages

Combining these modules produces consistent gains in both detection accuracy and model efficiency. When Sonar-DualNet and GLSA-BiFPN are used together, the model becomes more compact, while the mAP50 and mAP50:95 increase to 0.889 and 0.533, respectively. The complete architecture, which incorporates all three modifications, achieves the best overall performance with an mAP50 of 0.896 and an mAP50:95 of 0.543. It also requires the fewest computational resources, reducing the parameter count to 11.38 M and the FLOPs to 23.7 G. These values correspond to reductions of approximately 42.7% and 58.4%, respectively, compared with the baseline. The ablation results show that combining the proposed changes in the backbone, neck, and encoder improves detection performance while substantially reducing computational cost. This balance is particularly important for pipeline defect detection in sonar images, where targets are often difficult to distinguish because of the low signal-to-noise ratio.

**Figure 10:**
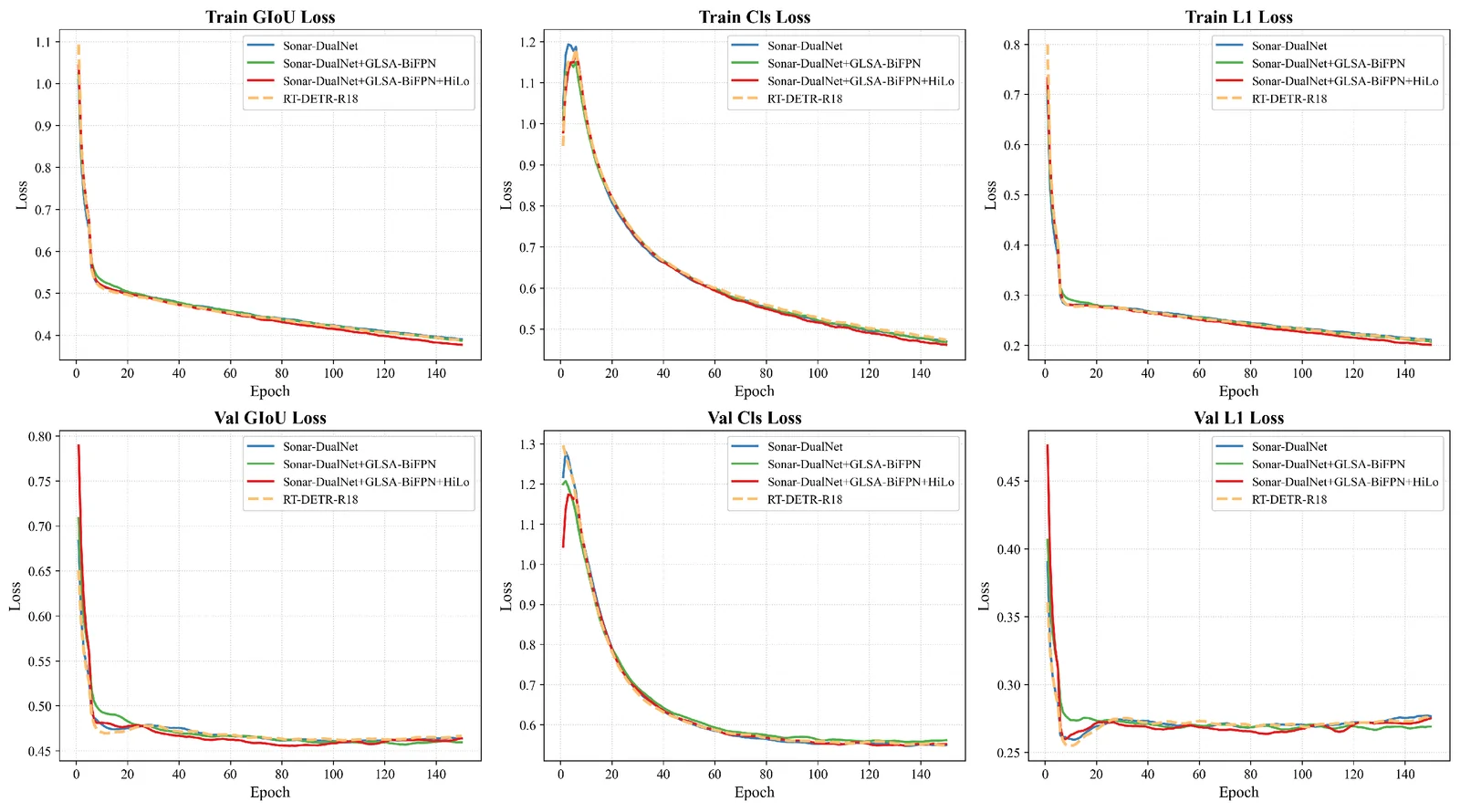
Training and validation loss curve comparison between LSD-DETR and baseline models.

Figure 10 shows the loss curves of LSD-DETR and the baseline models on the training and validation sets, including GIoU loss, L1 loss, and Cls loss. For all four models, the losses decrease rapidly during the early stages of training and then gradually converge with no clear signs of divergence or overfitting. Compared with the baseline models, represented by the light orange dashed lines, the complete model (Sonar-DualNet + GLSA-BiFPN + HiLo) consistently achieves lower values across all three loss terms. The lower training losses suggest that the proposed model fits the training data more effectively, while the corresponding validation curves indicate stable generalization throughout training.

**Figure 11:**
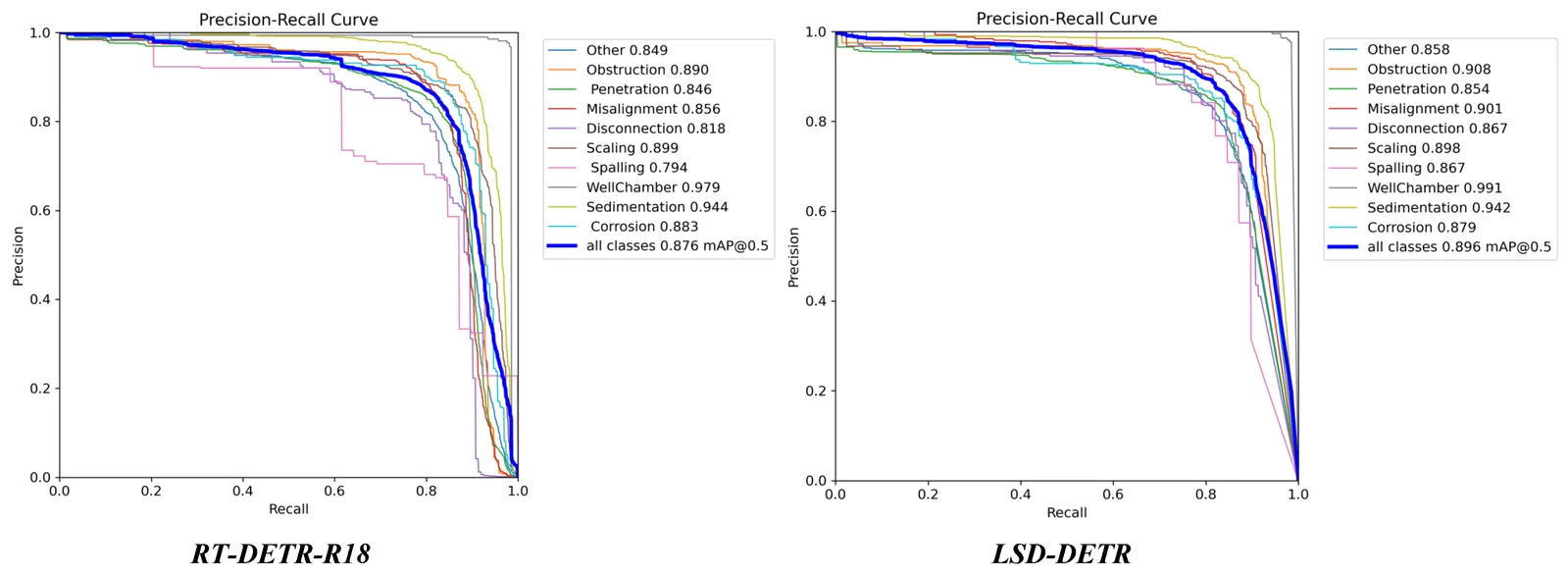
Validation set Precision–Recall (P-R) curve comparison between LSD-DETR and baseline models.

Figure 11 shows the Precision–Recall (P-R) curves of LSD-DETR and the baseline models on the validation set. LSD-DETR achieves a consistently higher and smoother P-R curve with an mAP50 of 0.896. This result indicates that the model maintains high precision across a broad range of recall values. The improvement is particularly evident for small targets with weak features, suggesting that LSD-DETR is more effective at detecting challenging sonar defects.

**Figure 12:**
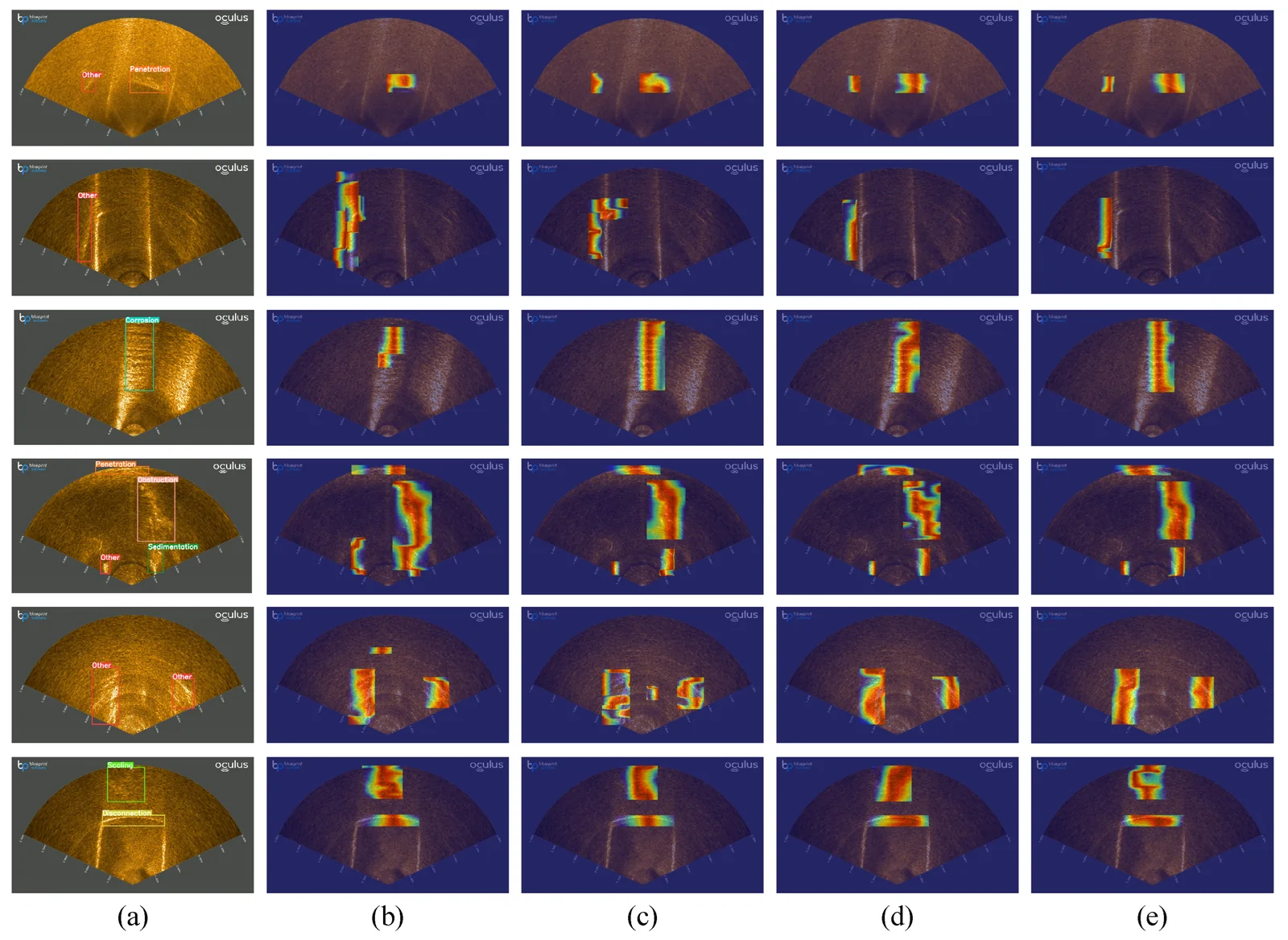
Comparison of Grad-CAM heatmaps on the test set between RT-DETR and its improved variant: (**a**) ground-truth annotation; (**b**) RT-DETR; (**c**) Sonar-DualNet; (**d**) Sonar-DualNet + GLSA-BiFPN; and (**e**) Sonar-DualNet + GLSA-BiFPN + HiLo (LSD-DETR).

To compare the performance, feature distributions, and regions of interest across different architectures for sonar defect detection, we use Grad-CAM++ to generate heatmaps for RT-DETR and its improved variants, as shown in Figure 12. The heatmaps indicate which regions of the sonar images contribute most strongly to the predictions of each model and thus provide insight into their feature responses during defect detection. Compared with the baseline, the improved models attend to broader and more accurately localized defect regions, suggesting a stronger ability to capture relevant features.

This difference is particularly evident in the fourth-row example, which contains multiple obstacle defects. LSD-DETR identifies both complex and small-scale anomalies more reliably, focusing on the relevant regions while producing fewer false positives and false negatives. These results show that LSD-DETR is better suited to detecting multiple objects and low-saliency targets in sonar images.

To assess the contribution of each component, we conduct fine-grained ablation experiments by replacing individual modules in the complete LSD-DETR architecture while keeping the remaining settings unchanged. The quantitative results are reported in Table 3. Replacing HGStem with standard CBS convolutions reduces mAP50 by 1.3%, suggesting that HGStem contributes to both detection accuracy and target localization. Replacing DR-Fusion with simple feature concatenation causes the largest performance drop across the evaluation metrics, although the parameter count decreases to 8.86 M and the FLOPs to 20.6 G. This result indicates that DR-Fusion introduces additional computational cost but provides substantial gains in detection performance, making it an important component of the overall architecture. Replacing GLSA with standard convolutions also degrades performance across all metrics. This finding suggests that the global spatial attention mechanism in GLSA captures contextual information that is not sufficiently represented by standard convolutions, thereby improving the model’s overall feature representation.

As shown in Table 4, to clarify whether the gain of DR-Fusion simply comes from generic adaptive weighting, we further compare it with both AFF and a Softmax-normalized variant under the same LSD-DETR configuration.

All variants retain the same backbone, neck, encoder, detection head, and training protocol. DR-Fusion-Softmax differs from DR-Fusion only by replacing the independent sigmoid gates with branch-wise Softmax normalization. AFF improves over direct concatenation, confirming that adaptive fusion itself is beneficial. However, DR-Fusion further increases mAP50/mAP50:95 from 0.883/0.531 to 0.896/0.543. More importantly, replacing its independent sigmoid gates with branch-wise Softmax normalization reduces the performance to 0.891/0.537 while leaving the model complexity almost unchanged. This result indicates that the improvement is not solely due to introducing attention-based fusion, but it is associated with the specific non-competitive gating formulation of DR-Fusion, which allows the two heterogeneous branch responses to be modulated independently rather than being forced to compete under a unit-sum constraint. These results indicate that the proposed fusion formulation is better suited to the heterogeneous representations produced by Sonar-DualNet.

### 5.2. Comparative Experiments

#### 5.2.1. Backbone Network Comparison Experiment

For comparative evaluation, this paper tests several typical lightweight backbone networks and their variants; the quantitative results are presented in Table 5. Replacing the original ResNet18 backbone with these alternatives leads to varying degrees of performance degradation for most models. Although EfficientViT, FasterNet, and MobileNetV4 reduce computational complexity, they also show noticeable losses in detection accuracy, suggesting that their feature extraction mechanisms are less well suited to sonar imagery. In comparison, Sonar-DualNet provides a better balance between efficiency and detection performance. It reduces FLOPs and the parameter count by 40.4% and 29.6%, respectively, while maintaining an mAP50:95 comparable to that of the baseline. It also achieves the highest precision and mAP50 among all evaluated backbones. These results indicate that Sonar-DualNet can extract effective features from sonar images with substantially lower computational and parameter costs than the other lightweight alternatives.

#### 5.2.2. Comparison of Feature Fusion Networks

Table 6 compares the baseline CCFM module with several feature fusion architectures for pipeline defect detection in sonar images. The results show that most lightweight fusion strategies reduce computational cost at the expense of detection accuracy. In contrast, the proposed GLSA-BiFPN achieves a better trade-off between efficiency and accuracy. Compared with CCFM, GLSA-BiFPN reduces the FLOPs by approximately 13.9% to 49.1 G and decreases the parameter count by 11.0%. It also improves the mAP50 to 0.882, the highest value among the evaluated configurations, while maintaining the same mAP50:95 as the baseline. BiFPN performs bidirectional cross-scale feature fusion and uses learnable weights to adapt the contributions of features from different scales. At each scale, GLSA combines global and local spatial attention to refine the fused features. This design allows the network to capture both the overall structure of pipelines and fine-grained defect patterns. The resulting feature representation helps mitigate the information loss associated with model compression and improves the detection of densely distributed defects and targets with unclear boundaries.

#### 5.2.3. Comparison of Attention Modules

Table 7 compares the baseline AIFI module with several encoder attention mechanisms for pipeline defect detection. Replacing AIFI with AIFI-DAttention or FDT does not improve detection performance: the mAP50 decreases to 0.870 and 0.865, respectively, together with a clear reduction in mAP50:95. These results suggest that general-purpose attention mechanisms may be less effective in sonar images, where complex background noise can interfere with feature representation. In comparison, HiLo increases the mAP50 to 0.880, the highest value among the evaluated configurations, while reducing the parameter count and keeping the computational cost close to that of the baseline. This improvement can be attributed to its dual-path attention design. The local-window branch retains fine-grained spatial interactions, whereas the pooled-global branch captures long-range context using fewer key and value tokens. Overall, this combination of local and global attention provides a better trade-off between detection accuracy and model complexity than the other attention mechanisms considered.

#### 5.2.4. Comparative Evaluation of LSD-DETR and Mainstream Baseline Models

This section presents a comparative evaluation against two-stage region proposal methods, alongside mainstream single-stage and Transformer-based detectors, establishing a comprehensive performance baseline on the SCTD and UPSD datasets. Table 8 reports the quantitative results for the LSD-DETR architecture and several representative baseline models on the UPSD dataset; Table 9 details the corresponding performance on the SCTD dataset. These comparisons evaluate the adaptability and robustness of the proposed approach across diverse scenarios and task conditions.

Analysis of the baseline evaluations on the UPSD dataset, as presented in Table 8, yields several key conclusions.

LSD-DETR achieves a recall of 0.849, an mAP50 of 0.896, and an mAP50:95 of 0.543, all of which are the highest among the evaluated models. Compared with the RT-DETR-R18 baseline, recall increases from 0.817 to 0.849, mAP50 from 0.876 to 0.896, and mAP50:95 from 0.527 to 0.543, corresponding to gains of 3.2, 2.0, and 1.6 percentage points, respectively. Precision decreases slightly from 0.874 to 0.865, but the higher recall indicates that LSD-DETR identifies a larger proportion of annotated defects and produces fewer missed detections under the same evaluation protocol. Compared with YOLOv12s, LSD-DETR also achieves higher recall, mAP50, and mAP50:95, although its precision is lower. The main advantage of LSD-DETR therefore lies in its average precision and defect retrieval rather than in precision alone. These results suggest that the heterogeneous backbone, adaptive feature fusion, and local–global attention mechanism jointly contribute to improved detection performance for sonar pipeline defects.

LSD-DETR also achieves a substantial reduction in model complexity. Relative to RT-DETR-R18, the parameter count decreases from 19.88 M to 11.38 M, while FLOPs decrease from 57.0 G to 23.7 G, corresponding to reductions of 42.8% and 58.4%, respectively. Under the reported batch-size-one setting, the inference speed increases from 59.5 FPS to 90.2 FPS. These results show that the proposed architecture improves detection accuracy without relying on a larger model or higher computational cost. Among the evaluated Transformer-based detectors, LSD-DETR has the lowest parameter count and FLOPs and achieves the highest measured inference throughput. Compared with RT-DETR-R34, it reduces the parameter count and FLOPs by 63.7% and 73.3%, respectively, while improving both mAP50 and mAP50:95. Although several compact one-stage detectors retain advantages in parameter count, FLOPs, or inference speed, LSD-DETR provides stronger overall detection accuracy. Therefore, its main contribution lies in achieving a favorable accuracy–complexity trade-off rather than being the smallest or fastest detector among all compared models.

To further examine whether the proposed architecture can be applied to another sonar detection dataset, all compared models are trained and evaluated separately on SCTD using the stated experimental protocol. Because the target-domain training labels are used, this experiment evaluates cross-dataset applicability. The background acoustic scattering characteristics within this dataset diverge significantly from the UPSD pipeline scenario, posing a substantial challenge to the cross-domain generalization of detection architectures. Table 9 summarizes the quantitative comparisons against representative baseline models. When trained and evaluated separately on SCTD, LSD-DETR achieves an mAP50 of 0.754 and an mAP50:95 of 0.480. These results demonstrate that the proposed architecture remains applicable to a second sonar detection task and provides a competitive accuracy–complexity trade-off under dataset-specific training.

**Figure 13:**
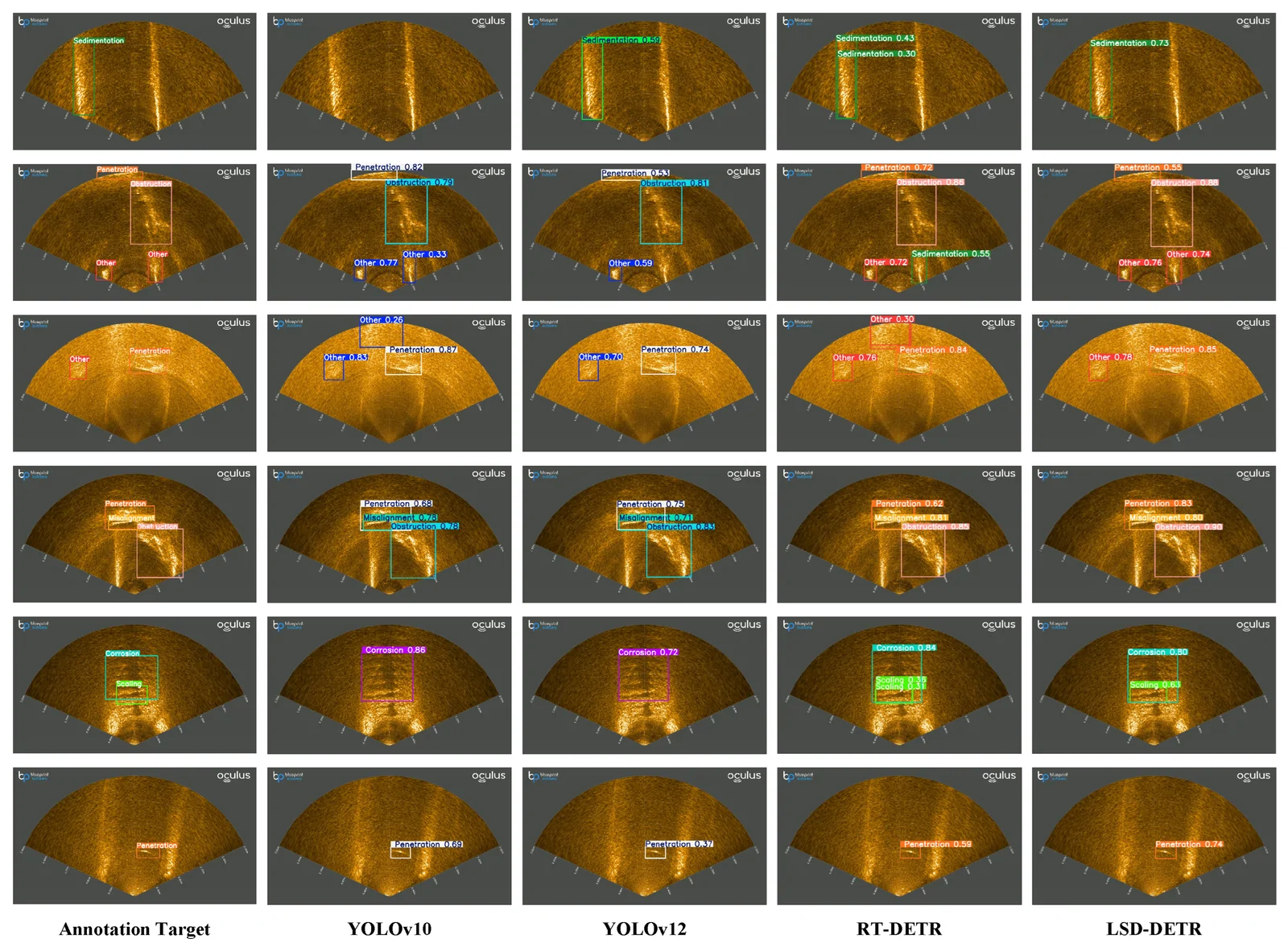
Visual comparison of detection results on the test set between the LSD-DETR model and mainstream baseline models.

Figure 13 compares the detection results of LSD-DETR with those of representative baseline models on the sonar defect test set. The first column shows the manually annotated ground truth, while the remaining columns present the predictions of the evaluated models. Compared with the baselines, LSD-DETR produces bounding boxes that align more closely with the ground truth and detects a larger proportion of the target defects. The qualitative results suggest that LSD-DETR is less affected by background noise in underground sewer sonar images, leading to more accurate target localization and more reliable defect detection.

Although most baseline models detect the more prominent defects successfully, their performance degrades in more complex scenes. In the first row, which contains Sedimentation, and the fifth row, where Corrosion and Scaling occur together, YOLOv10 and YOLOv12 miss several defects, while RT-DETR produces redundant bounding boxes or less accurate localization. Similar limitations are observed in the second and fourth rows, which contain densely distributed defects from multiple categories, including Penetration, Misalignment, Obstacles, and other small-scale targets. In these cases, the baseline models have difficulty separating closely spaced defects, resulting in false positives or overlapping predictions across categories. LSD-DETR detects low-saliency small targets, including defects in the Other category, more reliably and provides clearer boundaries for overlapping defects. These qualitative results suggest that the proposed model is better able to handle multiple, subtle, and closely spaced defects in cluttered sonar images, improving detection reliability in complex underground sewer environments.

## 6. Discussion

The results support the working hypothesis that heterogeneous feature extraction, adaptive branch fusion, and local–global contextual modeling can improve sonar-based sewer-defect detection without increasing model complexity. Compared with RT-DETR-R18, LSD-DETR improves mAP50 from 0.876 to 0.896 and mAP50:95 from 0.527 to 0.543 while reducing the parameter count from 19.88 M to 11.38 M and FLOPs from 57.0 G to 23.7 G. These results indicate that the accuracy improvement is not achieved by enlarging the network. Instead, the shared-stem heterogeneous backbone reduces duplicated shallow computation while preserving complementary feature representations, thereby producing a more favorable accuracy–complexity trade-off.

The improvements can be interpreted in relation to previous sewer-pipe and sonar detection studies, which commonly enhance conventional detectors through lightweight backbones, feature pyramids, or attention mechanisms. Sonar-DualNet differs from a standard lightweight backbone by organizing existing CSP/C2f-based and HGNetV2-based blocks into two complementary paths with shared shallow processing. DR-Fusion further performs content-conditioned fusion using independent spatio-channel gates and branch-specific channel scaling rather than relying on fixed addition, direct concatenation, or normalized competition. Meanwhile, the GLSA-enhanced BiFPN and HiLo modules strengthen bidirectional cross-scale interaction and separate local-window modeling from pooled-global contextual modeling. The results therefore suggest that jointly improving feature diversity, adaptive fusion, and multi-scale context is beneficial for detecting weak, noisy, and scale-varying defects in water-filled underground sewer pipelines.

The reported RTX 4090 throughput of 90.2 FPS under the batch-size-one setting demonstrates efficient desktop-GPU inference, but it should not be interpreted as direct evidence of deployment on an embedded pipe-inspection platform. Similarly, the SCTD result of 0.754 mAP50 demonstrates applicability to a second sonar dataset because the model was trained and evaluated separately on that dataset rather than direct cross-domain generalization. The remaining false positives and missed detections in complex backgrounds, overlapping defects, and structurally ambiguous regions indicate that further improvements are still required. Future research should therefore investigate the multimodal fusion of sonar and CCTV data, broader training data covering different pipe materials and wastewater conditions, direct cross-device transfer, and deployment-oriented evaluation on actual sewer-inspection hardware.

## 7. Conclusions

This paper develops LSD-DETR, which is a parameter-efficient RT-DETR-R18-based detector for forward-looking sonar defect detection in water-filled underground sewer pipelines. Its principal paper-specific designs are Sonar-DualNet, which organizes existing CSP/C2f-based and HGNetV2-based blocks into a shared-stem heterogeneous backbone, and DR-Fusion, which combines input-dependent spatio-channel gating with branch-specific channel scaling. Existing BiFPN, GLSA, and HiLo mechanisms are further incorporated as task-oriented neck and encoder adaptations.

On the UPSD dataset, LSD-DETR achieves an mAP50 of 0.896 and an mAP50:95 of 0.543, improving the RT-DETR-R18 baseline by 2.0 and 1.6 percentage points, respectively. Meanwhile, the parameter count and FLOPs are reduced by 42.7% and 58.4%, outperforming the baseline RT-DETR and several state-of-the-art detectors. These results demonstrate that LSD-DETR improves sonar-based sewer-defect detection while maintaining a compact computational profile, thereby providing a favorable accuracy–complexity trade-off for the automated condition assessment of water-filled underground sewer pipelines.

## Figures and Tables

**Table 1:** Specifications of the Oculus MD750D.

Parameter Name	Value
Operating frequency	750 kHz/1200 kHz
Measuring range (large)	100 m/40 m
Measuring range (small)	0.1 m
Distance resolution	4 mm/2.5 mm
Update rate	40 Hz
Horizontal beam opening angle	130°/80°
Vertical beam opening angle	20°/12°
Number of beams	512
Angular resolution	1°/0.6°
Beam spacing	0.25°/0.16°

**Table 2:** Ablation experiment results for each module.

Base	Sonar-DualNet	GLSA-BiFPN	HiLo	FLOPs (G)	Params (M)	mAP50	mAP50:95
✓				57.0	19.88	0.876	0.527
✓	✓			34.0	13.99	0.886	0.526
✓		✓		49.1	17.69	0.882	0.527
✓			✓	57.1	19.85	0.880	0.524
✓	✓	✓		25.9	11.78	0.889	0.533
✓	✓	✓	✓	23.7	11.38	0.896	0.543

Note: A checkmark (✓) indicates that the corresponding module is included in the model configuration.

**Table 3:** Fine-grained ablation studies on key component designs. Each row compares a modification with the complete LSD-DETR model.

Component	Modification	FLOPs (G)	Params (M)	${\mathrm{mAP}}_{50}$	${\mathrm{mAP}}_{50:95}$
Full Model	Baseline for comparison	23.7	11.38	0.896	0.543
w/o HGStem	Replace HGStem with CBS	24.1	11.21	0.883	0.532
w/o DR-Fusion	Replace DR-Fusion with Concat	20.6	8.86	0.878	0.526
w/o GLSA	Replace GLSA with standard convolution	24.3	11.21	0.883	0.528

**Table 4:** Comparison of heterogeneous feature fusion strategies within the complete LSD-DETR frameworks.

Fusion Method	FLOPs (G)	Params (M)	mAP50	mAP50:0.95
Concat	20.6	8.86	0.878	0.526
AFF [18]	20.8	13.50	0.883	0.531
DR-Fusion-Softmax	23.9	11.39	0.891	0.537
DR-Fusion	23.7	11.38	0.896	0.543

**Table 5:** Comparison results using different backbone networks.

Backbone	FLOPs (G)	Params (M)	P	R	mAP50	mAP50:95
ResNet18 [38]	57.0	19.88	0.874	0.817	0.876	0.527
EfficientViT [39]	27.3	10.71	0.872	0.807	0.873	0.520
FasterNet [40]	28.8	10.90	0.851	0.808	0.861	0.513
FasterNet-EMA [41]	51.5	16.91	0.871	0.837	0.883	0.530
FasterNet-CGLU [42]	46.2	15.41	0.863	0.810	0.872	0.513
MobileNetV4 [43]	39.5	11.32	0.856	0.799	0.863	0.516
Sonar-DualNet	34.0	13.99	0.884	0.826	0.886	0.526

**Table 6:** Comparison of different feature fusion modules.

Method	FLOPs (G)	Params (M)	P	R	mAP50	mAP50:95
CCFM [21]	57.0	19.88	0.874	0.817	0.876	0.527
Slimneck [44]	53.3	19.31	0.882	0.820	0.880	0.525
BiFPN [34]	47.7	17.23	0.863	0.826	0.878	0.519
SSFF [45]	46.0	9.02	0.854	0.808	0.867	0.513
WFU [46]	46.8	16.82	0.867	0.836	0.877	0.513
GLSA-BiFPN	49.1	17.69	0.868	0.829	0.882	0.527

**Table 7:** Comparison of different attention modules.

Method	FLOPs (G)	Params (M)	P	R	mAP50	mAP50:95
AIFI [21]	57.0	19.88	0.874	0.817	0.876	0.527
AIFI-DAttention [47]	57.2	19.89	0.864	0.820	0.870	0.519
DTAB [48]	58.7	21.76	0.870	0.850	0.875	0.528
FDT [46]	57.5	20.23	0.878	0.804	0.865	0.507
HiLo	57.1	19.85	0.863	0.831	0.880	0.524

**Table 8:** Performance comparison of different models on the UPSD dataset.

Model	FLOPs (G)	Params (M)	P	R	mAP50	mAP50:95	${\mathrm{FPS}}_{\left(\mathrm{bs}=1\right)}$
Two-stage Object Detectors							
Faster R-CNN [10]	208.0	41.39	0.768	0.763	0.823	0.479	16.8
Cascade R-CNN [49]	236.0	69.80	0.778	0.753	0.814	0.475	25.7
One-stage Object Detectors							
YOLOv8n [50]	8.7	2.80	0.833	0.825	0.882	0.526	144.5
YOLOv9t [51]	7.6	1.97	0.826	0.785	0.850	0.514	113.8
YOLOv10s [52]	21.4	7.22	0.855	0.829	0.891	0.538	142.3
YOLOv12s [53]	19.3	9.07	0.883	0.824	0.893	0.541	119.0
Transformer-based Object Detectors							
DETR [20]	96.5	41.56	0.801	0.806	0.819	0.468	25.3
DAB-DETR [54]	102.0	43.70	0.752	0.753	0.795	0.445	20.5
Conditional-DETR [55]	101.0	43.45	0.860	0.808	0.864	0.495	22.4
Deformable-DETR [56]	193.0	40.10	0.837	0.781	0.840	0.474	11.1
RT-DETR-R18 [21]	57.0	19.88	0.874	0.817	0.876	0.527	59.5
RT-DETR-R34 [21]	88.8	31.32	0.890	0.824	0.881	0.533	51.4
LSD-DETR (Ours)	23.7	11.38	0.865	0.849	0.896	0.543	90.2

**Table 9:** Performance comparison of different models on the SCTD dataset.

Model	FLOPs (G)	Params (M)	P	R	mAP50	mAP50:95
Two-stage Object Detectors						
Faster R-CNN [10]	208.0	41.39	0.812	0.620	0.736	0.470
Cascade R-CNN [49]	236.0	69.80	0.840	0.605	0.702	0.476
One-stage Object Detectors						
YOLOv8n [50]	8.7	2.80	0.708	0.561	0.671	0.408
YOLOv9t [51]	7.6	1.97	0.877	0.622	0.706	0.433
YOLOv10s [52]	21.4	7.22	0.827	0.520	0.644	0.411
YOLOv12s [53]	19.3	9.07	0.667	0.641	0.712	0.426
Transformer-based Object Detectors						
DETR [20]	96.5	41.56	0.518	0.575	0.551	0.357
DAB-DETR [54]	102.0	43.70	0.479	0.611	0.529	0.298
Conditional-DETR [55]	101.0	43.45	0.723	0.626	0.715	0.435
Deformable-DETR [56]	193.0	40.10	0.657	0.650	0.732	0.433
RT-DETR-R18 [21]	57.0	19.88	0.861	0.682	0.722	0.467
RT-DETR-R34 [21]	88.8	31.32	0.731	0.718	0.746	0.481
LSD-DETR (Ours)	23.7	11.38	0.777	0.803	0.754	0.480

## Data Availability

The original contributions presented in this paper are included in the article. Further inquiries can be directed to the corresponding author.
